# Lessons learnt while designing and conducting a longitudinal study from the first Italian COVID-19 pandemic wave up to 3 years

**DOI:** 10.1186/s12961-023-01055-w

**Published:** 2023-10-31

**Authors:** Alvisa Palese, Stefania Chiappinotto, Federico Fonda, Erica Visintini, Maddalena Peghin, Marco Colizzi, Matteo Balestrieri, Maria De Martino, Miriam Isola, Carlo Tascini

**Affiliations:** 1 Department of Medicine (DAME), University of Udine, Udine, Italy; 2 Infectious and Tropical Diseases Unit, Department of Medicine and Surgery, University of Insubria-ASST-Sette Laghi, Varese, Italy; 3 Unit of Psychiatry, Department of Medicine (DAME), University of Udine, Udine, Italy; 4 Department of Psychosis Studies, Institute of Psychiatry, Psychology and Neuroscience, King’s College London, London, United Kingdom; 5 Medical Statistics Division, Department of Medicine (DAME), University of Udine, Udine, Italy; 6 Infectious Diseases Division, Department of Medicine (DAME), University of Udine, Udine, Italy

**Keywords:** COVID-19, Longitudinal study, Research design, Lessons learned

## Abstract

**Background:**

Several scientific contributions have summarized the “lessons learnt” during the coronavirus disease 2019 (COVID-19) pandemic, but only a few authors have discussed what we have learnt on how to design and conduct research during a pandemic. The main intent of this study was to summarize the lessons learnt by an Italian multidisciplinary research group that developed and conducted a longitudinal study on COVID-19 patients infected during the first wave in March 2020 and followed-up for 3 years.

**Methods:**

A qualitative research approach embedded into the primary CORonavirus MOnitoRing study (CORMOR) study was developed, according to the the consolidated criteria for reporting qualitative research. Multiple data collection strategies were performed: each member was invited to report the main lessons learnt according to his/her perspective and experience from the study design throughout its conduction. The narratives collected were summarized and discussed in face-to-face rounds. The narratives were then thematically analysed according to their main topic in a list that was resent to all members to check the content and their organization. The list of the final “lessons learnt” has been agreed by all members, as described in a detailed fashion.

**Results:**

Several lessons were learnt while designing and conducting a longitudinal study during the COVID-19 pandemic and summarised into ten main themes: some are methodological, while others concern how to conduct research in pandemics/epidemics/infectious disease emergencies.

**Conclusions:**

The multidisciplinary approach, which also included patients’ perspective, helped us to protect the consistency and quality of the research provided in pandemic times. The lesson learnt suggest that our research approach may benefit from changes in education, clinical practice and policies.

**Supplementary Information:**

The online version contains supplementary material available at 10.1186/s12961-023-01055-w.

## Background

The several lessons learnt during the coronavirus disease 2019 (COVID-19) pandemic in clinical practice, in addition to the management of wards and entire hospitals/health care services, as well as in the understanding of the disease itself, constitute an important knowledge for all those who have lived this difficult professional experience. Transforming this rich heritage of individual and collective experience into structured knowledge, to be handed over to future generations, rendering them able to face future challenges effectively and resiliently, is also an ethical imperative [1–3]. In this context, several scientific contributions have summarised the “lessons learnt” during the difficult COVID-19 period, in different fields such as education [4], clinical and managerial [5] ones: as a result, more than 4000 papers are retrievable in PubMed when the search string “lessons learnt” and “COVID-19” is applied. However, despite the unprecedented number of studies published to date [6], which changed also the impact factors of journals [7], a few have discussed the lessons learnt on conducting research during a pandemic. The risk of disruption to some research lines due to restrictions (for example, [8]) and researchers’ redeployment has been debated [9]. Violations in the protocols designed before the pandemic have also been investigated in their influence on the quality of the research [10]. How to ensure data collection online instead of face to face [11] was discussed to ensure continuity in some studies. In addition, how to identify community-relevant research questions during pandemics [12] as well as the consequences for doctoral/resident students who were unable to continue their studies [13–15] have been analysed. Previous outbreaks have been a useful resource for addressing the current one [16], which has been characterized by an urgent need to generate knowledge and immediately disseminate it to mitigate and control the pandemic and its devastating effects [17]. In this context, the aim of this contribution is to summarize the lessons learnt by a multidisciplinary research group that designed and conducted a longitudinal study starting in March 2020, which included patients affected by the first wave of COVID-19.

### The coronavirus monitoring (CORMOR) longitudinal study

As well known, Italy was among the first countries hit by the pandemic, with the north regions, where our centre is located, being dramatically affected since the beginning of March 2020 when all the world was unprepared to face the pandemic. In this context, a group of clinicians fully engaged in the care of patients since the first cases, immediately designed a longitudinal project, named CORonavirus MOnitoRing study (CORMOR), aimed at describing the serological evolution among COVID-19 patients over time [18–23], integrating clinical data. Over time, the project has produced knowledge in vaccination hesitancy [24] and long-term neuropsychiatric sequelae [25, 26], as well as the overall experiences of patients [27, 28] and the strategies enacted by nurse managers in nursing homes to prevent the spread of the virus [29] (Additional file 1). Therefore, within the longitudinal quantitative nature of this study design, qualitative data collections were nested according to the emerging research needs.

**Fig. 1:**
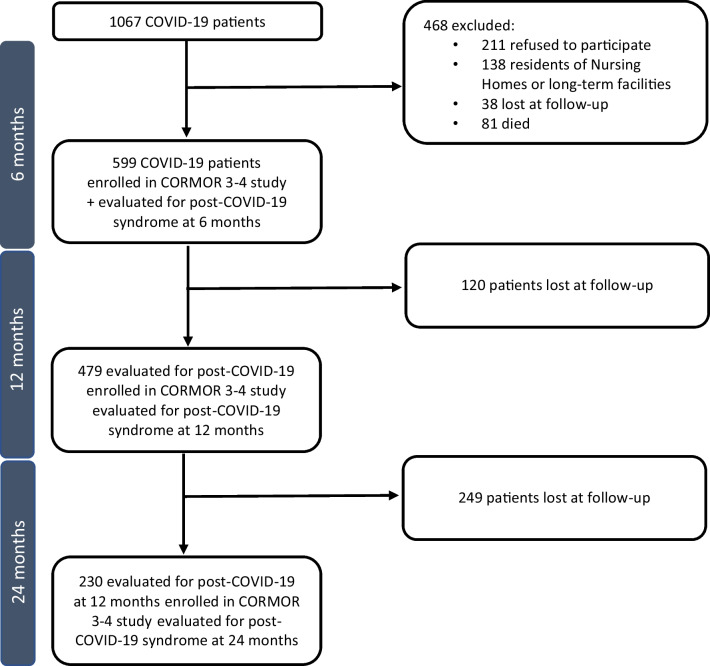
Flow diagram of in- and outpatients with COVID-19 included in the studies. COVID-19: coronavirus disease 2019; CORMOR 3–4: CORonavirus MOnitoRing Study parts 3 and 4

The target population was composed of patients who were (a) adults (≥ 18 years old); (b) cared for by the infectious disease referral centre of an academic hospital located in the northeast serving as a point of reference for about 500 000 citizens; (c) in- and outpatients (for example, day hospital); and (d) diagnosed as having confirmed or suspected COVID-19 from March to May 2020 [30]. The target was composed of 1067 patients; among them, 439 were excluded, resulting in 599 patients (Fig. 1). Data were collected at the following times: (a) at the COVID-19 diagnosis, as well as after 6, 12 and 24 months. Namely, at the disease onset (a) sociodemographic (for example, gender) and (b) clinical data (for example, severity of COVID-19) were all collected and recorded in a database. The severity of the COVID-19 disease was categorized into asymptomatic, mild, moderate, severe and critical disease [30]. At 6 months, and at the following data collection points, an interview based upon open- and closed-ended questions from a piloted instrument was conducted to collect data regarding, among other aspects, symptoms and experiences of patients over time. The project was approved by the regional ethics committee (CEUR-2020-OS-219 and CEUR-2020-OS-205).

## Methods

### Study design and theoretical framework

A qualitative research approach embedded into the primary CORMOR study was developed and is reported here according to the the consolidated criteria for reporting qualitative research (COREQ) [31] as summarized in the Additional file 2. The methodological orientation of this research exercise was based on the value of reflection [32]. In fact, Schon underlined that the practical knowledge of each action is central to the work of practitioners. This form of knowledge, called “knowing-in-action”, is nurtured by the “reflection-in-action” as the active and non-propositional processes by which new knowledge is developed while doing an action [32]. Therefore, the theoretical propositions of Schon [32] regarding the value of the reflection-in-action process as one capable to generate new knowledge, were considered as a reference point for this research exercise.

### Research setting and participants

While carrying out the CORMOR study, regular meetings were conducted to check the quality of the data collection, discuss and interpret the findings, and provide further lines of investigation. During these meetings, findings were also discussed and reflections on the research process itself were performed. After around 1 year, the original research team was expanded by including additional members according to the complexity of an unfolding phenomenon needing multiple perspectives. Therefore, all members of the multidisciplinary team, composed of senior and junior researchers and practitioners, appertaining to different disciplines (see Additional file 3), were involved in an additional research exercise aimed at identifying the lesson learnt: all agreed to participate in the proposed exercise.

### Data collection and analysis

At the end of the second year, an in-depth reflection [33] was initiated, aiming at collecting multiple data to be merged into the “lessons learnt”, while designing and conducting a longitudinal study in difficult times such as the pandemic was. At the beginning, the intent was presented and agreed upon by all members of the team, also in terms of data collection procedures. Then, the procedures were established as follows: First, each member was invited to report in a written format the main lessons learnt according to his/her perspective and experience from the study design conception since its implementation. The open-ended question was: “What lesson did you learn during this longitudinal study?”. No specific guidelines were provided to allow the maximum richness and freedom in providing the answer. The invitation was sent to all, with 2 weeks for individual reflection and reporting. Only one reminder was sent to ensure that all members provided their feedback. Second, all narratives sent by members were collected by one senior research (AP) and then first approached with two researchers (SC, FF). One comprehensive narrative synthesis was composed, including all data collected from the team members, by scrutinizing the similarities and differences across the narratives received [34]. Then, a thematic analysis [35] was performed using an inductive approach [36]: each provided lesson was independently analyzed by three researchers (AP, SC, FF) and then agreed upon.

The first draft produced a clear description of the lessons learnt, also reporting the quotes of participants in an anonymized fashion (for example, Participant 1). This was then resent to participants and subsequently enriched and discussed during a face-to-face meeting with all. During such a meeting, lasting 1.5 hours, the draft was further shaped, and emerging in-the-field notes were collected by two researchers (SC, FF). Then, a third email round was undertaken by sending the revised draft including all changes suggested during the face-to-face meeting and based on the notes collected. The reflections were then further organized and categorized by three researchers (AP, SC, FF) and resent to all members to check the contents and their organization [37]. No other lessons emerged, thus suggesting saturation [38]. The list of the final “lessons learnt” was agreed by all members as described in a detailed fashion by including (a) their label, (b) a description and (c) a brief discussion of their meaning, also according to the literature available.

### Rigour

To ensure trustworthiness, the COREQ guidelines [31] were followed to perform each step; moreover, there was a prolonged engagement in the research exercise, lasting more than 1 year. In addition, emerging lessons were member checked [37] and the saturation [38] was discussed by all members of the team [37].

## Findings and discussion

Several lessons were learnt while designing and conducting a longitudinal study during the COVID-19 pandemic. The lessons that participants reported have been summarized under ten main themes (Table 1): some are methodological, while others concern how to conduct a research study in pandemics/epidemics/infectious disease emergencies.

**Table 1:** Designing and conducting a longitudinal study from the first Italian COVID-19 pandemic wave up to 3 years: the main lessons learnt

1. Leading and accelerating research processes in an uncertain scenario
2. Broadening the research aims based on the emerging literature, as well as by listening to patients and reflecting on our daily practice
3. Preventing the progressive selection of patients and thinking about the still-neglected populations
4. Challenges in engaging patients in the research process
5. Discerning findings as individual or family/dyad perceptions
6. Transforming a longitudinal study into a surveillance and caring service
7. Adapting the data collection methods over time to the emerging needs/issues to balance time, ensure efficiency and protect research integrity
8. Experiencing the interesting challenge of combining quantitative and qualitative data
9. Should the focus be on the first wave or on the subsequent one as well?
10. Being cared for and making sense of troubled times

### Leading and accelerating research processes in an uncertain scenario

Before 2020, our way of approaching research projects was traditional, based on certain background information and on knowledge gaps to be filled with equally certain information to collect, but also on stable and accurate diagnostic systems, in a method of conducting research that was substantially comfortable, known and based on reflection and (more or less) the availability of time. Research related to COVID-19 was instead rapid, designed on uncertain bases, on many or few hypotheses, and often fluctuating; in addition, it was triggered by urgent needs that required immediate answers, in which the interoperability of electronic systems was often not yet available [39]. It was stressful and maximized our capacity as clinical researchers to intercept new and interesting hypotheses by bringing together clinic and laboratory data, as well as data concerning the experience of patients and that of health care workers, in a continuing process of triangulation. Data analysis was also expected to be performed immediately to render available and disseminate the knowledge gained. No one was prepared for this impetuous way of doing research and the involvement of young colleagues such as postgraduates and residents was necessary for learning purposes, but also because of the substantial research workloads. We learnt to accelerate the research processes, mainly thanks to our closeness to the patient, which was always inspiring and provided insights, as well as the capacity to provide immediate data analysis and write reports, and the invaluable support of the multidisciplinary team that was open to new members to understand an unfolding phenomenon and the patients’ emerging issues.

### Broadening the research aims based on the emerging literature, as well as by listening to patients and reflecting on our daily practice

The CORMOR study was originally designed to follow the clinical and serological response of patients: data on infections caused by respiratory viruses, in general, are limited in the current literature. In fact, most of the serological data available have epidemiological and serological responses for evaluation purposes related to the vaccine response to the influenza virus. In this context, it was extremely important to have a strong clinical background given that many clinical and microbiological aspects of the disease and post-COVID-19 effects were potentially intuitable and based on the experience related to Middle East respiratory syndrome (MERS) and severe acute respiratory syndrome coronavirus 1 (SARS-CoV-1). Over time, other aspects emerged as important, including, for example, the role of reinfection: are patients who have already had the COVID-19 disease at risk of being reinfected? Considering the atypicality of SARS-CoV-2 and of the associated inflammatory and cytokine response, could this reinfection be more or less severe than the first episode? Therefore, the typical laboratory and clinical research was enriched, and a more public health approach was adopted by investigating aspects related to the vaccination. In the same way, visiting patients who were followed for serological follow-ups triggered new ideas in researchers listening to their complaints on the long-lasting persistence of symptoms. In the following stages, this study focused on the need to increase knowledge regarding the relevance of the neuropsychological/neuropsychiatric implications of the post-COVID-19 syndrome. In summary, an open and flexible research process was designed, where variables were progressively reviewed. However, such an approach sometimes affected the opportunity to compare the collected data with previously gathered information. During the research project, the team also incorporated competences which did not initially appear essential (for example, psychiatric). Therefore, how to balance the expectations of each discipline regarding the data to collect to avoid an excessive burden on patients was also learnt.

### Preventing the progressive selection of patients and thinking about the still-neglected populations

Our study included all patients who were referred to our infectious disease unit during the first pandemic wave, which was in an academic hospital and functioned as a reference point for around 500 000 citizens. With the progression of restrictions related to the pandemic, especially during the lockdown, the selection of the population gradually increased. Specifically, while at the beginning of the wave a heterogeneous group of patients who were working, living or transiting in our region were cared for, with the lockdown, our centre (and consequently our study) gradually started to care only for local patients. This risk of selection bias, which is a threat in all studies, thus increased its relevance because of the restrictions imposed. At the overall level, all studies were exposed to a tremendous stress and uncertainty due to the exceptional circumstances. Developing alliances with other infectious disease centres, to build up a network (for example, 40) could have ensured not only more heterogeneity in the patients included, but also the strengthening of the study robustness.

Moreover, as our flow diagrams shows (Fig. 1), we were unable to include residents living in nursing homes. Nursing homes became immediately inaccessible due to the severe restrictions imposed [41]; the same data collection by telephone or by video call would have been complex and not feasible due to the extreme difficulty for nursing staff in managing daily care. Moreover, the data collection from cognitively declined residents could not be carried out remotely and would have required special relational strategies to be conducted face to face, which was also unfeasible in this case. Furthermore, collecting informed consent from family, who were anxious about what was happening in nursing homes and not easily reachable, was a challenge. Our cohort failed to include nursing home residents, who represent a major population of infected patients which has probably faced the worst consequences, and which requires more attention in the future.

### Challenges in engaging patients in the research process

Patients affected by COVID-19 in the first wave were initially very intimidated by the disease and grateful to the clinicians and the whole health care system. Therefore, they were very cooperative and their engagement in the research process was very easy. They also listened carefully during each research step, that was – in some ways – co-constructive given the value attributed to their complaints and experiences in the follow-up visits. By listing their symptoms that informed new research questions, they actively contributed to the research development itself. However, over time, this study faced a certain degree of loss to follow-up, in line with any other research projects [42], as well as an increased risk of attrition bias, as elderly individuals or those with comorbidities, for example, were more affected by the COVID-19 disease. Differently, family members were substantially excluded from the research process, given the limitations in the hospital policies [43]. Their point of view may be at merit for consideration in futures studies.

### Discerning findings as individual or family/dyad perceptions

Since the source of the infection was predominantly within the family [44], more individuals were involved in our study from the same family. With reference to the more subjective variables, such as emotions and experiences, we still have doubt as to whether patients reported what they perceived and experienced as individuals or, instead, as a dyad (for example, elderly couples) or a family. The role of significant relationships in ways of living and coping with the disease is well reported in the literature [45, 46]. There may have been differences between those who, alone in the disease, faced it without a supporting person, and those who experienced their disease together with their family, which was also the source of the contagion. Developing strategies to disentangle, deepen and compare the individual perceptions with that of dyads or families, from the perspective of “emotional clusters” would be very interesting.

### Transforming a longitudinal study into a surveillance and caring service

In the longitudinal data collection, we involved expert nurses who called the patients to perform the data collection. Despite the number of attempts, it was possible to reach only a proportion of eligible patients through telephone contacts (Fig. 1). However, by calling patients, the research group ensured an active surveillance and caring strategy towards them, identifying critical situations, which were detected and referred to the infectious disease or mental health centres. Specifically, out of 479 patients of the first wave interviewed at 1 year, one patient was referred to the infectious disease centre, while after 2 years, out of the 230 patients contacted, 11 were referred to the infectious disease centre and three to the mental health centre. Among these patients, who were forced to face long waiting lists to access the health care system, the active surveillance provided by our research group, performed through telephone calls, facilitated the early identification of at-risk issues, which otherwise would not have been identified. Therefore, our research process also functioned as an active surveillance system. However, when patients with evident emotional suffering were interviewed and refused to be referred to the mental health centre, research nurses were morally distressed by the dilemma of deciding whether to (a) report the individual anyway, especially when they were alone without any family support, therefore violating their confidentiality; or (b) respect the preference of the patient and protect their privacy. We faced this challenge by providing these individuals with more calls, protecting their privacy, ensuring the required support and trying to facilitate the first contact with the specialized centres. These additional calls where not in line with the research protocol but ensured a kind of caring service and support.

### Adapting the data collection methods over time to the emerging needs/issues to balance time, ensure efficiency and protect research integrity

The first questionnaire used for the 6 month data collection was significantly expanded at 1 and 2 years, including psychological/psychiatric dimensions according to the emerging knowledge on post-COVID-19 mental health issues. This lengthened the telephone administration of the questionnaire, from the initial 20 min to 40–60 min for each interviewee, potentially discouraging participants. This problem prompted the researchers to find alternative data collection strategies to meet the needs of the participants, by using a virtual platform (EUSurvey) that was accessible at any time. The introduction of two methods of data collection (based on calls and the web), using the same questionnaire according to the preference of patients, made it possible to (a) optimize the data collection in terms of adherence for those patients who were short on time and (b) increase efficiency from the research team’s perspective. However, in implementing this decision, three key lessons have been learnt:

- *Making a first call to all*. All patients first received a call at each time point of the longitudinal study. This was important to establish a connection, maintain engagement and simply ask “How are you after 6 months?” Then, the best strategy to collect data (via phone or the web) was decided with them. If the data collection had been based solely on the web-based questionnaire, the value of the telephone-based direct contact with patients, in terms of expressing active surveillance and involvement in deciding the best way to collect data, would have been lost. We learnt the importance of performing data collection according to the preferences of patients.
- *Preventing low participant rates and bias*. The web-based questionnaire was effective for the digitally competent patients, who filled in the questionnaire on demand and independently. Therefore, setting up both methods of data collection made it possible to reach all eligible patients with an inclusive approach, as well as ensured good response rates.
- *Piloting different methods to ensure accuracy and reliability*. Data collection might be influenced by the methods used, and what might be simple to collect via the web (for example, filling in a Likert scale based on seven points), might be a challenge during a phone interview where the multiple options available may challenge the patients. On the other hand, expressing emotions via the web might be difficult, whereas during a phone interview the competencies of the researchers may facilitate the reporting of experiences. All these aspects might have introduced an information bias. We did not perform a pilot phase to assess whether participants answered differently when using the web- or the phone-based data collection methods. A pilot phase before launching the questionnaire could have contributed to the understanding of the comparability of the data collected with both methods (for example, intrarater reliability).

### Experiencing the interesting challenge of combining quantitative and qualitative data

In our research tradition, the (comfortable) tendency was to describe either experiential, clinical or biological data, by using different interpretative quantitative-based research paradigms. In our study, we combined the biological, clinical and experiential data, prompting methodological challenges and advancements. On one hand, different and more refined knowledge regarding the diagnosis and treatment of the disease has been established over time; on the other, this process made it particularly complex to interpret or consider valid the data within the cohort, as, for example, in the case of mental health issues, where psychometric tools are considered the gold standard for assessment of common mental disorders in the general population.

### Should the focus be on the first wave or on the subsequent one as well?

This is still an open question inside the research team. Our study involved patients who were affected by the virus in the first wave. Continuing to pay attention to this cohort through a longitudinal study has required resources, meaning that less research attention has been given to other patients in the following waves, who have developed different forms of the disease due to different virus variants. Undoubtedly, deepening the exploration into the issues experienced by the first patients was important in developing the knowledge regarding this severe and unprecedented phenomenon. However, the research resources dedicated to these patients were higher than those received by those diagnosed in the second and following waves, when it was not sustainable to ensure a follow-up due to resource constraints.

### Being cared for and making sense of troubled times

We experienced difficult times during the pandemic, with several discouraging moments and issues that all had long-term effects. Those of us who were immersed in the clinical arena did not see the light for several months due to the lockdown, the hard work, the fear of the contagion, the number of patients who died and other aspects all well documented in the literature [47]; those of us who were outside of the clinical arena perceived a sense of uselessness. We cannot hide that conducting research in these hard times has had a sort of “antidepressant” effect: we persisted in our hope to contribute to the resolution of the problem with new data, as well as projecting our mind beyond the everyday COVID-19 issues. Moreover, by working together we supported each other, by ensuring a space to perform research to clinical members directly involved in the care of patients. This experience expanded our capacity to do research.

### Limitations

This research exercise had several limitations. First, the data collection process was conducted with limited guidance/standardization: despite ensuring the maximum variations and freedom to participants, this may have introduced some bias in data collection and reporting. Secondly, although the narratives were blinded and thus anonymized, the reflections of senior researchers may have influenced that of junior ones, especially during the online meetings, thus introducing also, in this case, a source of bias. Third, the data analysis was conducted by the same researchers involved in the data collection; they were all part of the CORMOR team, thus with previous well-established relationships. While this may have enriched the process, given their deep knowledge of the study, on the other hand they may have influenced the analysis according to their background. Fourth, the data collection was performed as a “reflection-in-action” according to the theoretical orientation of this research exercise; other lessons may have been learnt if developed as a “reflection-on-action”, retrospectively contemplating the whole research experience at the very end [48]. In addition, the categorization process resulting in the ten main lessons learnt was performed by a single team; beyond these lessons, several other learnings may have been gained during the last 3 years of personal and professional experience if gathered outside the CORMOR study, capable of influencing both the design and conduction of future research.

## Conclusions

A multidisciplinary approach to research has always been conceptualized but its implementation has been slow. However, such a need was pragmatically applied in our experience, by nurturing the same research process with the contribution not only of the team, which has been expanding in terms of members, but also (almost) of the patients themselves. In fact, the contributions of colleagues with different clinical and research backgrounds, as well as different stakeholders, helped us to protect the consistency and the quality of the research provided during the pandemic. This reflective-based and transversal approach taught us strategies and issues that will certainly be remembered in our future research projects.

The lessons learnt suggest that our research approach may benefit from changes in education, clinical practice and policies, to be more effective in facing critical events as the one lived. At the educational level, there is a need to better prepare future scientists that are ready to confront themselves with the need to investigate issues in pandemic times. In this perspective, to promote flexible attitudes, to sustain the capacity to be open to the insights and emerging needs of patients, to prevent any rigid approach in clinical practice and to promote specific strategies to avoid loose ends (for example, the risk to neglect some populations), are some examples that should be considered (for example, in doctorate programmes). At the organizational level, it is recommended to promote work health care environments capable of supporting an effective research team (for example, providing time for multidisciplinary research). At the policy level, strategies to prioritize research, to create networks facilitating cooperation across facilities and to move away from a “reactive research model” to a “proactive” one, are suggested. In general, there is a need to promote multidisciplinary approaches capable of enriching our perspectives that are prerequisites while investigating complex issues.

### Supplementary Information

**Additional file 1.** Publications and main aims.

**Additional file 2.** Consolidated criteria for reporting qualitative studies (COREQ): 32-item checklist.

**Additional file 3.** Multidisciplinary team.

## Data Availability

This is a methodological paper. The datasets used and/or analysed during the mentioned studies are available from the corresponding author on reasonable request.

## Abbreviations

COVID-19: Coronavirus disease 2019
CORMOR: CORonavirus MOnitoRing study
MERS: Middle East respiratory syndrome
SARS-CoV-1: Severe acute respiratory syndrome coronavirus 1
SARS-CoV-2: Severe acute respiratory syndrome coronavirus 2
