# Supplementary material for: Lessons learnt while designing and conducting a longitudinal study from the first Italian COVID-19 pandemic wave up to 3 years
Source: Health Res Policy Syst. 2023 Oct 31;21:111. doi: 10.1186/s12961-023-01055-w (PMC10617212; doi:10.1186/s12961-023-01055-w)
Supplement: Supplementary file 1 — Additional file 1. Publications and main aims. [file 12961_2023_1055_MOESM1_ESM.docx]

**Additional file 1.** Publications and main aims.

| **Reference** | **Aims** | **Citations**^†^ |
| --- | --- | --- |
| Peghin et al. (20) | To assess the prevalence of and factors associated with post-coronavirus disease 2019 (COVID-19) syndrome 6 months after the onset. | 133 |
| Peghin et al. (19) | To assess reinfection rates in relation to long-term antibody dynamics against SARS-CoV-2 after the first wave. | 10 |
| Peghin et al. (22) | To assess the long-term dynamics and factors associated with the serological response against the severe acute respiratory syndrome coronavirus 2 after primary infection. | 25 |
| Gerussi et al. (24) | To assess the attitude towards influenza and severe acute respiratory syndrome coronavirus 2 (SARS-CoV-2) vaccinations among coronavirus disease 2019 (COVID-19) recovered patients. | 55 |
| Peghin et al. (21) | To describe the impact of vaccination and the role of humoral responses on post-COVID-19 syndrome 1 year after the onset of SARS coronavirus type 2 (CoV-2). | 16 |
| Colizzi et al. (25) | To investigate the potential development of a post-COVID mental health syndrome in the longer-term and identify its risk factors. | 12 |
| Peghin et al. (18) | To  (a) compare the efficacy of SARS-CoV-2 antibody detection between RDT and laboratory serology, trying to identify appropriate semi-quantitative cut-offs for RDT in relation with quantitative serology values and  (b) evaluate diagnostic accuracy of RDT compared to the NAAT gold standard in an unselected adult population. | 3 |
| Palese et al. (27) | To understand the entire experience of patients regarding the COVID-19 disease; to describe the emotional orientation (positive, neutral, or negative) of the metaphors expressing their lived experience. | 1 |
| Palese et al. (29) | To identify interventions implemented during the first, second and third waves of the COVID-19 pandemic among Italian Nursing Homes. | 1 |
| Palese et al. (28) | To collect ongoing COVID-19 survivors’ pandemic-related experiences as expressed through the use of metaphors and to explore socio-demographic variables associated with the metaphor orientation as negative, positive or neutral. | 0 |
| Colizzi et al. (26) | To raise attention on the need for neuropsychiatric support by prospectively assessing the occurrence of mental-health-domain-related symptoms over a 24-month period following COVID-19 onset in a cohort of 230 patients | 0 |
| Peghin et al. (23) | The describe the long-term evolution of post-COVID-19 syndrome over 2 years after the onset of severe acute respiratory syndrome coronavirus type 2 (SARS-CoV-2) in survivors of the first wave. | 0 |

^†^ Citations according to Scopus, retrieved on the 27th of August 2023.

**Legend**. SARS-CoV-2: Severe Acute Respiratory Syndrome CoronaVirus 2; COVID-19: Coronavirus Disease 2019; RDT, Rapid Diagnostic Tests; NAAT, Nucleic Acid Amplification Test.
