# Supplementary material for: Lessons learnt while designing and conducting a longitudinal study from the first Italian COVID-19 pandemic wave up to 3 years
Source: Health Res Policy Syst. 2023 Oct 31;21:111. doi: 10.1186/s12961-023-01055-w (PMC10617212; doi:10.1186/s12961-023-01055-w)
Supplement: Supplementary file 3 — Additional file 3. Multidisciplinary team. [file 12961_2023_1055_MOESM3_ESM.docx]

**Additional file 3**. Multidisciplinary team.

| **Team** | **Background/education** | **Main Field** | **Experience in research** |
| --- | --- | --- | --- |
| Peghin Maddalena, *Female* | Medical Doctor, PhD | Infection Diseases | Senior |
| Carlo Tascini, *Male* | Medical Doctor | Infection Diseases | Senior |
| Marco Colizzi, *Male* | Medical Doctor, PhD | Psychiatry | Senior |
| Matteo Balestrieri, *Male* | Medical Doctor, PhD | Psychiatry | Senior |
| Miriam Isola, *Female* | Statistician | Statistics | Senior |
| Maria De Martino, *Female* | Statistician, PhD candidate | Statistics | Intermediate |
| Stefania Chiappinotto, *Female* | Registered Nurse, PhD | Nursing | Intermediate |
| Federico Fonda, *Male* | Registered Nurse, MNSc | Nursing | Junior |
| Erica Visintini, *Female* | Registered Nurse, MNSc | Nursing | Junior |
| Alvisa Palese, *Female* | Registered Nurse, PhD | Nursing | Senior |

**Legend**. PhD, Doctor of Philosophy; MNSc, master’s degree in nursing and Midwifery Sciences candidate.
